# Interplay of mitochondrial calcium signalling and reactive oxygen species production in the brain

**DOI:** 10.1042/BST20240261

**Published:** 2024-08-22

**Authors:** Plamena R. Angelova, Andrey Y. Abramov

**Affiliations:** Department of Clinical and Movement Neurosciences, UCL Queen Square Institute of Neurology, London, U.K.

**Keywords:** calcium, glial cell, mitochondria, mitochondrial permeability transition pores, neuron, reactive oxygen species

## Abstract

Intracellular communication and regulation in brain cells is controlled by the ubiquitous Ca^2+^ and by redox signalling. Both of these independent signalling systems regulate most of the processes in cells including the cell surviving mechanism or cell death. In physiology Ca^2+^ can regulate and trigger reactive oxygen species (ROS) production by various enzymes and in mitochondria but ROS could also transmit redox signal to calcium levels via modification of calcium channels or phospholipase activity. Changes in calcium or redox signalling could lead to severe pathology resulting in excitotoxicity or oxidative stress. Interaction of the calcium and ROS is essential to trigger opening of mitochondrial permeability transition pore — the initial step of apoptosis, Ca^2+^ and ROS-induced oxidative stress involved in necrosis and ferroptosis. Here we review the role of redox signalling and Ca^2+^ in cytosol and mitochondria in the physiology of brain cells — neurons and astrocytes and how this integration can lead to pathology, including ischaemia injury and neurodegeneration.

## Introduction

Cells in the body are in constant intercommunications and to enable this they continuously transmit extracellular or intracellular signals. In excitable cells, such as neurons of the central nervous system, signal transduction is highly specialised and could be activated only by specific neurotransmitters which trigger a signalling cascade. However, so called ‘second messengers’ are designed to be universal. Thus, the calcium ion (Ca^2+^) regulates almost all processes in the cells –all what happens with the organism and its cells from fertilisation, division, metabolism to cell death. This universality of Ca^2+^ signalling properties is based on the intracellular compartmentalisation of Ca^2+^ pools: low Ca^2+^ concentration in the cytosol and more than 10 000× higher Ca^2+^ concentration outside the plasma membrane [1], as well the existence of intracellular Ca^2+^ storage in several organelles such as the endoplasmic reticulum and the mitochondria, for example. Upon stimulation, plasmalemmal or intracellular Ca^2+^-channels trigger calcium signal which varies by amplitude and localisation. There are a number of Ca^2+^-conducting channels in neurons including ionotropic glutamate receptors, store-operated Ca^2+^ channels, voltage-sensitive calcium channels, etc. which open in response to a stimulus and allow Ca^2+^ to enter into the cytosol. Metabotropic receptors are initiating a cascade of reactions that leads to the generation of IP3 or ryanodine, which in turn activates the receptors on the endoplasmic reticulum to release Ca^2+^ along the concentration gradient into the cytosol [2].

The calcium gradient is restored by various Ca^2+^/other ion exchangers but mainly by the action of Ca^2+^-ATPases on the plasma membrane (PMCA) and endoplasmic reticulum (SERCA). The maintenance of intracellular Ca^2+^ homeostasis is highly energy-dependent process and it cost to the cells a major part of the total intracellular ATP [3]. Mitochondria are key elements in the mechanism of calcium signalling, playing the role of a short-term calcium buffer [4]. Mitochondrial calcium uptake via the mitochondrial calcium uniporter is electrogenic and dependent on the mitochondrial membrane potential. At the same time Ca^2+^ uptake activates mitochondrial dehydrogenases that stimulate respiration and ATP production [5]. Ca^2+^-efflux in mitochondria of brain cells is operated by Na^+^/Ca^2+^ exchanger [6] molecularly identified as NCLX [7].

Living in atmosphere of oxygen has adapted most of the organisms to this otherwise toxic gas and made them vitally dependent on its presence. Despite the tight isolation of the brain from the rest of the body, i.e. the existence of brain blood barrier which makes delivery of various biological molecules and gases more complicated, the major brain cells — neurons and astrocytes consume more oxygen than the cells from any other tissue. This could be explained by their extremely high energy demand to enable transmission of the signal and electric activity of the neurons, as well as restoration of the resting potential which consumes most of the cellular ATP. Molecular oxygen is active, but its partially reduced forms are thousands of times more active due to asymmetry of their molecular electron density or the presence of unpaired electron which leads to the formation of free radical. However, together with different types of true oxygen radicals, including superoxide (O_2_^•−^) and the hydroxyl radical (OH^•^), the term reactive oxygen species (ROS) includes oxygen non-radical forms — O_3_, hydrogen peroxide (H_2_O_2_) and singlet oxygen ^1^O_2_ that are not free radicals *per se*, but easily converted into free radicals [8,9]. ROS have different reactivity and lifetime that define their involvement in physiological and pathological processes. ROS are produced exogenously (by light or radiation) and enzymatically in various cells including neurons and glia. Thus, ROS are not only a by-product of enzymatic reactions — even in places such as the electron transport chain (ETC) of mitochondria, where superoxide or hydrogen peroxide are produced continuously. Considering the extremely high activity and toxicity of ROS, cells and particularly postmitotic neurons which live long lives, must be in possession of a very efficient antioxidant system. More importantly, the intensity of ROS production also depends on the metabolic rate and various other conditions that can be used by the cell as a signalling event [10].

**Figure 1.**
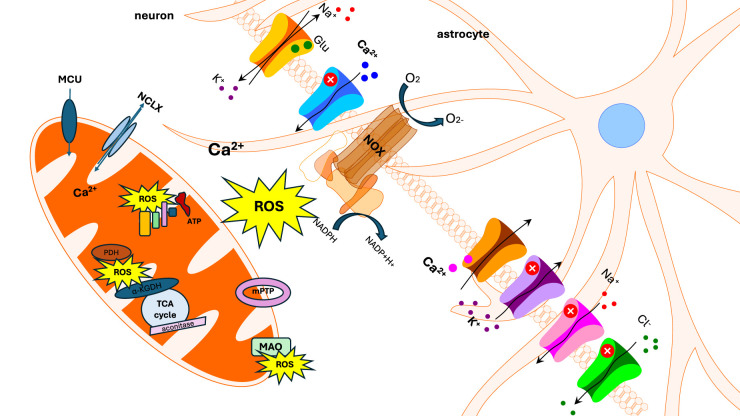
Interaction of the mitochondrial Ca^2+^ and redox signalling in brain cells. ROS-reactive oxygen species, MAO, monoamine oxidase; TCA cycle, tricarboxylic acid cycle; PDH, pyruvate dehydrogenase; α-KGDH, α-ketoglutarate dehydrogenase; ATP, adenosine triphosphate; MCU, mitochondrial calcium uniporter; NCLX, mitochondrial sodium/calcium exchanger; NOX, NADPH oxidase; NADPH, nicotinamide adenine dinucleotide phosphate.

Ca^2+^ could translate signal in ROS through activation of mitochondrial metabolism and production of superoxide in ETC [11,12] (Figure 1), in neurons and astrocytes calcium signal which is triggered by glutamate or purinergic receptor could activate ROS production in NADPH oxidase [13–15]. And *vice versa* — ROS and the products of oxidation could activate signalling event through Ca^2+^ in neurons and astrocytes [16,17] that could also lead to elevation of mitochondrial calcium.

Deregulation of the calcium homeostasis or ROS-induced oxidative stress has been shown to be the triggering step in the mechanism of multiple brain pathologies including stroke and neurodegeneration [18,19].

Mitochondria are organelles which are responsible for multiple functions in the cells including energy production and regulation of cell death/survival. Importantly, both Ca^2+^ and ROS are triggers for the opening the mitochondrial permeability transition pore (mPTP) — the initial step of cascade mechanism of cell death [20] (Figure 1).

## Ca^2+^-induced production of ROS in neurons and astrocytes

Mitochondrial Ca^2+^ signal and concentration are dependent on several factors:

(a) Mitochondrial calcium uptake is dependent on extramitochondrial Ca^2+^ (in cytosol and endoplasmic reticulum through MEM (mitochondrial-reticulum contacts) and can be elevated only if calcium in the cytosol is elevated [4,21].
(b) Mitochondrial calcium uptake via MCU activity (mitochondrial calcium uniporter) is electrogenic and dependent on the mitochondrial membrane potential (Δψm) which in turn is dependent on the activity of the ETC [22].
(c) Transport of Ca^2+^ and inorganic phosphate inside the mitochondrial matrix are coupled, parts of Ca^2+^ and phosphate are stored as osmotically inactive precipitates. Mitochondria in brain cells have high capacity to accumulate and store Ca^2+^, most of which is in a bound form; estimates of the bound/free ratio can be up to 4000 [23,24].

Changes in mitochondrial calcium reflect changes in cytosolic calcium signal and the activation of mitochondrial dehydrogenases by Ca^2+^ which lead to the activation of ATP production for compensation of the energy consumed for restoration of [Ca^2+^]_c_ [25].

Production of ROS in ETC of mitochondria is dependent on the Δψm [26,27]. Mitochondrial calcium uptake induces short term mitochondrial depolarisation due to the charge of Ca^2+^ that is compensated by the activation of ETC and induces hyperpolarisation [21]. This in turn induces short term and small amplitude increase in ROS production in mitochondrial ETC. Thus, calcium uptake in mitochondria could transfer signal to redox changes that can be used second messenger for activation of the intracellular processes [10].

In the matrix of mitochondria ROS are produced during several enzymatic reactions in the tricarboxylic acid cycle (TCA) using aconitase, pyruvate dehydrogenase and α-ketoglutarate dehydrogenase [28]. Activity of these enzymes is dependent on Ca^2+^ concentration [29] and considering this, mitochondrial Ca^2+^ uptake can stimulate the generation of ROS in the TCA cycle, but nevertheless, overactivation may have inhibitory effect [30].

Calcium signal in both neurons and astrocytes is activated via specific receptors which are represented by the glutaminergic system in neurons and P_2_Y receptors in astrocytes. Interestingly, glutamate-induced calcium signal activates NADPH oxidase in both neurons [14] and astrocytes [13]. Glutamate-induced calcium signal, mediated by ROS, is used by cells for the maintenance of redox homeostasis but on the same time plays an important role in the development of pathology of ischaemia reperfusion injury, including CO-induced neurotoxicity [15,31] and also in β-amyloid toxicity in AD [32].

## ROS induce calcium signal in neurons and astrocytes

Monoamine oxidase A (MAO A) and monoamine oxidase B (MAO B) enzymes located on the outer mitochondrial membrane both play important and essential role in the homeostasis of catecholamines [33] (Figure 1). Both, MAO A or MAO B are utilising monoamines and produce hydrogen peroxide and aldehydes as by-products of the reaction. However, application of dopamine or adrenaline and utilisation of these monoamines in astrocytic MAO induces hydrogen peroxide production and consequent lipid peroxidation. This leads to the activation of phospholipase C, production of IP_3_ and ultimately triggers calcium signal [17,34]. Importantly, this receptor-independent adrenaline-induced calcium signal in astrocytes is responsible for changes of blood vessel diameter [17], that regulates brain oxygenation in physiology and deregulation of this process may lead to oxygen deprivation in brain regions and several pathologies, including dementia [35,36].

The ability of phospholipase C to be activated by oxidised lipids [16] is also established for the mechanism of physiological response to ischaemia. Decrease of oxygen level leads to a decrease in Δψm in astrocytes that induces ROS production, lipid peroxidation, PLC activation and increase of calcium signal [10,11]. Importantly, disease-associated mutation in the phospholipase PLA2G6 changing the activity of this enzyme [37] and leads to lipid peroxidation [38] that induces alteration of calcium handling in neurons and astrocytes [39].

Mitochondrial hyperpolarisation under some brain pathologies, including frontotemporal dementia (MAPT 17 mutation), induces massive production of superoxide in the ETC of these cells [40]. This mitochondrial ROS overproduction specifically oxidises proteins responsible for the transport NMDA and AMPA receptors and as a result alters and elongates glutamate-induced calcium signal [41,42]. Importantly, mitochondrial overproduction of ROS in these neurons also inhibits mitochondrial NCLX and the calcium efflux from this organelle [43].

ROS do not only activate calcium signal but also modify it. Thus, cytosolic hydrogen peroxide is shown to be able to oxidase sarco(endo)plasmic reticulum Ca^2+^-ATPase (SERCA) protein and to inhibit the activity of this enzyme [44]. In agreement to that, in rotenone-induced model of Parkinson's disease in Drosophila mitochondrial ROS overproduction was associated with submaximal SERCA inhibition [45].

## Interaction of ROS and Ca^2+^ in triggering of mPTP

Found in isolated mitochondria this transient and fast increase of membranal permeability for quite some time was just a phenomenon without any link to physiology or pathology [46], which could be induced by a number of activators including the main ones — elevated Ca^2+^ and ROS [47–49]. However, the interest to mitochondrial permeability transition has dramatically increased after it was directly linked to the process of cell death [20,50–52].

Although opening of the mPTP is thought to be involved in physiological processes as part of the mitochondrial fast Ca^2+^ efflux [49], mPTP ‘flickering’ is easy to activate without calcium signal only by introduction of singlet oxygen [53].

However, opening of the mPTP in neurons and astrocytes is more of a pathological event than a regulator of brain function and is deeply involved in the mechanism of neurodegeneration [20]. Thus, inhibition of mitochondrial calcium efflux in familial forms of Parkinson's disease — PINK1 and LRRK2 mutations [54–56] leads to mitochondrial calcium overload and mPTP opening. Activation of physiological calcium signal with dopamine and further production of hydrogen peroxide in MAO lead to opening of mPTP and cell death [57]. Interestingly, the same mechanism of pathology development — inhibition of NCLX, mitochondrial calcium overload and mPTP opening after oxidative stress, is shown for tauopathy [43] (Figure 1), for Alzheimer's disease [58] and age-associated cognitive decline [59]. Considering this, restoration of NCLX function [55] or pharmacological or molecular inhibition of mitochondrial calcium uptake is protective in animal models of neurodegenerative diseases [60–62].

Misfolding and aggregation of proteins with physiological function lead to modification of their function or toxic gain-of-function that leads to neuronal cell death [63]. Thus, monomeric α-synuclein plays a role in synaptic transduction and binding to mitochondrial F0-F1-ATPsynthase that increases the efficiency of ATP production [64,65]. Oligomeric α-synuclein is able to form channels and induce cytosolic calcium signal [66] and in the same time is able to produce superoxide in the presence of transition metal ions [67–69]. Binding of oligomeric α-synuclein to F0-F1-ATPsynthase in the same way as monomers do, oxidises the subunits of this mitochondrial enzyme and in combination with elevated mitochondrial calcium opens the mPTP and leads to neuronal cell death via the mechanism of ferroptosis [69].

## Role of mitochondrial Ca^2+^ and ROS in activation of cellular enzymes

Poly(ADP-ribose)polymerase (PARP) is a DNA-repairing enzyme family, overactivation of which can alter energy metabolism due to NAD^+^ consumption and thus can function as a trigger for cell death [70]. PARP is activated by toxins and, expectably, by ROS and cytosolic Ca^2+^ [71]. However, activation of PARP was shown to be responsible for a profound mitochondrial depolarisation and energy deprivation in glutamate excitotoxicity [18,72]. Thus, energy deprivation in many pathologies is a direct result from substrate unavailability to ETC enzymes from either substrate underproduction or increased mitochondrial respiration with consequent ETC substrate overconsumption [73]. However, inhibition of the mitochondrial calcium uptake protects neurons against mitochondrial depolarisation and cell death, suggesting that mitochondrial Ca^2+^ and ROS are essential for PARP activation [60,74]. ROS and mitochondrial Ca^2+^ are also responsible for PARP activation in beta-amyloid neurotoxicity and sequestration of mitochondrial calcium uptake protect cells against mitochondrial depolarisation and cell death [61,75].

## Redox regulation of ion transport mechanisms in the brain

Ion channels and other ion transport mechanisms (receptors, exchangers, pumps) endow the maintenance of ion gradients on intracellular and plasmalemmal membranes, that enable intra- and intercellular signalling. On the other hand, ion channels are protein complexes that contain cysteine residues with highly reactive thiol groups. Thus, signalling properties of ROS are enabled through the process of reversible oxidation of SH-groups on the channel proteins. In early eighties Nicoterra, Orenius and colleagues observed a strict correlation between the depletion of protein sulfhydryl groups and loss of cell viability that was mediated by disruption of calcium homeostasis [76].

Redox modulation has been first described for Ca^2+^-dependent K^+^ channel in 1983 [77] (Figure 1). Later on, various ion channels and ion transport proteins have been found to be modulated by ROS [78]. Among the currents that are largely influenced by the cellular redox status are also the inward and outward K^+^ currents (Sham and Shaw types), ATP-sensitive K^+^ channels (K_ATP_) [79], and hyperpolarization-activated inward current (*I_h_*) [80]. Interestingly, reducing agents decrease the activity of the large-conductance, calcium-activated potassium channels (BK, also BK_Ca_, Slo, or MaxiK) and the ROS-induced effects are largely concentration- and tissue-type-dependent [79]. Specific oxidative modulation of Kv1.4, Kv4.2 by calcium-dependent second messenger activated ROS release has been demonstrated [81,82].

Despite the sparce literature, there is also an evidence that voltage-dependent anion-selective channels in the mitochondrial outer membrane [83], small Cl– (SCl) channel and other Cl– channels are also modulated by an oxidation-reduction mechanism [84].

The latter list of ion channels and transport mechanisms, regulated by redox reactions is not exhaustive and is constantly growing. Thus, the involvement of emerging pathways for the regulation of neuronal functions through redox reactions and the possibility to modulate pathological processes through redox reactions paves the way of novel treatments even where oxidative stress is not the primary issue.

### In conclusion

Mitochondrial calcium and redox signalling are in close communication with each other. The two independent signalling systems could activate each other in physiology in the interest of signal transduction and intracellular communications. However, mitochondrial Ca^2+^ and ROS could synergistically enhance the action of each other in conditions of pathology resulting in oxidative damage, opening of mPTP and activation of different mechanism of cell death — apoptosis, necrosis and ferroptosis.

## Perspectives

- Mitochondrial ROS and Ca^2+^ are responsible for intracellular signalling, regulation of energy production and triggering the cell death.
- Interaction of the mitochondrial ROS and Ca^2+^ has been precepted more as a pathological event with clear evidence for synergetic action in physiological processes.
- Study of the transformation of the signals and mechanisms of interactions of mitochondrial Ca^2+^ and redox signalling may help in understanding many processes including oxygen sensing, adaptation of the organism to stress and mechanisms of pathology of the central nervous system.

## Open Access

Open access for this article was enabled by the participation of University College London in an all-inclusive *Read & Publish* agreement with Portland Press and the Biochemical Society under a transformative agreement with JISC.

## Abbreviations

ETC: electron transport chain
MAO A: monoamine oxidase A
MAO B: monoamine oxidase B
mPTP: mitochondrial permeability transition pore
PARP: poly(ADP-ribose)polymerase
ROS: reactive oxygen species
TCA: tricarboxylic acid cycle
